# Plasma glycated CD59 (gCD59), a novel biomarker for the diagnosis, management and follow up of women with Gestational Diabetes (GDM) – protocol for prospective cohort study

**DOI:** 10.1186/s12884-020-03090-9

**Published:** 2020-07-18

**Authors:** D. Bogdanet, PM. O’Shea, J. Halperin, F. Dunne

**Affiliations:** 1 College of Medicine Nursing and Health Sciences, National University of Ireland Galway, Galway, Ireland; 2 Diabetic Day Centre, Galway University Hospital, Galway , Ireland; 3 Department of Medicine, Brigham and Women’s Hospital, Harvard Medical School, MA Boston, USA

**Keywords:** Gestational diabetes, biomarker, CD59

## Abstract

**Background:**

The prevalence of Gestational Diabetes (GDM) is rising and with it the number of mothers and children at risk of adverse outcomes. As treatment has been shown to reduce adverse events, it is imperative that we identify all at-risk pregnant women. In Ireland, the national standard of care is selective screening with a 2-hour 75 g oral glucose tolerance test (OGTT). Aiming for universal screening is of utmost importance but this is difficult given the length, the unfeasibility and impracticability of the OGTT. We aim to assess if the novel biomarker glycated CD59 (gCD59) is a suitable contender for the OGTT in identifying women with GDM.

**Methods:**

In this prospective cohort study, the study participants will be consecutive pregnant women at Galway University Hospital, Galway, Ireland. Samples for the plasma gCD59 biomarker will be taken together with routine bloods at the first antenatal visit, at weeks 24–28 at the time of routine 75 g OGTT, in trimester 3- and 12-weeks post-partum for women with GDM while having their routine post-partum 75 g OGTT. The constructed database will contain baseline information on each study participant, baseline laboratory data, follow-up laboratory data and pregnancy related outcomes. We aim to recruit a total of 2,000 participants over the project period and with a national GDM prevalence of 12–13%, we will have 240–260 subjects who meet OGTT criteria for GDM. Following regional prevalence, we expect to have 34–37 women who will develop either diabetes or pre-diabetes in the early post-partum period. The sensitivity and specificity of plasma gCD59 to predict the results of the OGTT will be assessed using nonparametric estimates of the receiver operating characteristic (ROC) curves and respective area under the ROC curve (AUROC).

**Discussion:**

A body of clinical and experimental evidence supports a link between the complement system, complement regulatory proteins, and the pathogenesis of diabetes complications.

Building on this research, our study plans to look at the plasma gCD59 capacity to classify pregnant women with normal or abnormal glucose tolerance but also to assess if plasma gCD59 can be used as an early predictor for GDM, for adverse pregnancy outcomes and/or post-partum glucose intolerance.

## Background

Gestational diabetes is a global epidemic that causes adverse maternal and infant outcomes and also identifies the mother-child pair at risk of future diabetes, obesity and cardiovascular disease [1–4]. Treatment of GDM reduces the incidence of adverse outcomes [5–7]. Selective screening for GDM with a 75 g OGTT is the current Irish national standard of care for all at-risk pregnant women without established diabetes [8]. However, universal screening is the gold standard and advocated by international bodies [9]. Universal screening is difficult to achieve currently because the standard OGTT has to be done fasting, is lengthy, cumbersome for the woman and the health service, and has poor reproducibility [10–12]. Globally researchers are working to identify biomarkers that may replace the OGTT and allow universal screening to become a reality.

Glycated CD59 (gCD59) is one such novel biomarker. CD59 is a complement regulatory protein that protects “self” cells from complement-mediated damage [13, 14]. In diabetes, CD59 is inactivated by non-enzymatic glycation forming gCD59. Plasma gCD59, is a soluble form of CD59 shed from cell membranes. CD59 is a widely distributed membrane-bound inhibitor of the cytolytic membrane attack complex (MAC) of complement[15]. CD59 functions by binding to C8 and/or C9 in the nascent MAC and interfering with C9 membrane insertion and polymerization. The protein, which is present in all cells, is anchored to the external surface of the membrane by a lipid tail. As such it is exposed to the extracellular fluid and the glucose levels in the extracellular fluids. A soluble form of CD59 shed from cell membranes is present in the circulation and urine. Inhibition of the terminal complement cascade is the only known function of CD59 and there is no evidence in the literature that the protein is a marker of inflammation or any other condition, or that significant variations occur in different human diseases.

Pregnancy constitutes a major challenge to the maternal immune system because it requires tolerance of foetal allo-antigens encoded by paternal genes; failure to tolerate, triggers foetal rejection as it does in transplanted organs. The complement system plays a critical role in transplant rejection; similarly, excessive complement activation in the placenta places the foetus at risk for growth restriction or death [16–18].

Therefore, it is not surprising that the foetus is protected from maternal immune responses by an array of mechanisms that include trophoblast expression of key complement regulatory proteins such as decay accelerator factor (DAF), membrane cofactor protein (MCP) and CD59. The key role played by complement and its regulators in pathological pregnancy is highlighted by experimental and clinical data showing that either immunologic maladaptation with activation of complement targeted to the placenta or decreased complement restriction contributes to the imbalance of angiogenic factor that is associated with placental dysfunction in preeclampsia [19–22]. Regarding pregnancy complications of diabetes, it is conceivable that glycation-inactivation of placental CD59 increases complement-mediated placental damage contributing in part to the multiple complications seen in women with GDM.

Plasma gCD59 can be measured with a highly sensitive and specific enzyme-linked immunosorbent assay (ELISA). Preliminary work in a US population screened by a 2-step glucose challenge test (GCT) followed by 100 g OGTT using Carpenter & Coustan criteria, shows promising benefits of plasma gCD59 as a biomarker for GDM [23]. This work demonstrated that plasma gCD59 is 8.5-fold higher in those with a positive compared to a negative GCT and 10-fold higher in those who had a positive OGTT following the positive GCT compared to those with a negative OGTT.

The purpose of this prospective study is to examine the validity of plasma gCD59 as a biomarker for the prediction, diagnosis, management and follow up of women with GDM diagnosed using the newer evidence based International Association of the Diabetes and Pregnancy Study Groups (IADPSG) criteria in a 1-step approach using a 75 g OGTT across all BMI categories in an unselected pregnant population. The IADPSG criteria confirm a diagnosis of GDM when fasting glucose is ≥ 5.1 mmol/L (92 mg/dl), 1-hour glucose is ≥ 10.0 mmol/L (180 mg/dl) or 2-hour glucose is ≥ 0.5 mmol/L (153 mg/dl) following a standard 75-g OGTT[1]. As only 1 test and 1 abnormal value are required when using IADPSG criteria, a greater spectrum of women with GDM will be identified. Thus, this study can explore plasma gCD59 in both lower risk and higher risk women.

## Methods/Design

### Study Design

#### Prospective cohort study

##### Objectives

1. To assess if levels of plasma gCD59 in early pregnancy predict GDM diagnosed by standard of care 75 g OGTT at weeks 24–28.
2. To assess if plasma gCD59 at weeks 24–28 can replace the 75 g OGTT as a diagnostic test for GDM.
3. To assess if plasma gCD59 levels in the course of pregnancy help monitor the effectiveness of treatment in women with GDM by examining pregnancy outcomes.
4. To assess if in women with GDM, second trimester (T2) and/or postpartum levels of plasma gCD59 can predict the conversion to diabetes/prediabetes as detected by a 75 g OGTT at 12 weeks post-partum.

Inclusion criteria

- Pregnant women 18 years old and over willing and able to provide informed consent.

Exclusion criteria

- Pregnant women with prior established diabetes.
- Women with concomitant disease or condition that, in the clinical judgment of the investigator, is likely to prevent the subject from conforming to the protocol.

### Recruitment

The study participants will be consecutive pregnant women booking for antenatal care at Galway University Hospital (GUH), Galway, Ireland. The patient information leaflet (PIL) (Supplementary material, ‘Patient information leaflet’) will be given at the first clinic appointment. The PIL will contain information on the study and a telephone number where the study participant can contact a member of the research team for questions. A member of the research team will explain the study purpose and methodology to the potential study participant. If agreeable, a consent form will be signed, and the first sample of blood will be taken at the time of routine first trimester bloods. The potential study participant will be informed and reassured regarding the low-risk of the study (the samples would be taken at the same time points as routine blood testing) and the confidentiality of the data collected. They will also be informed they can withdraw from the study at any point in time. It will be explained clearly that they can decline to participate without their care being affected in any way.

**Fig. 1:**
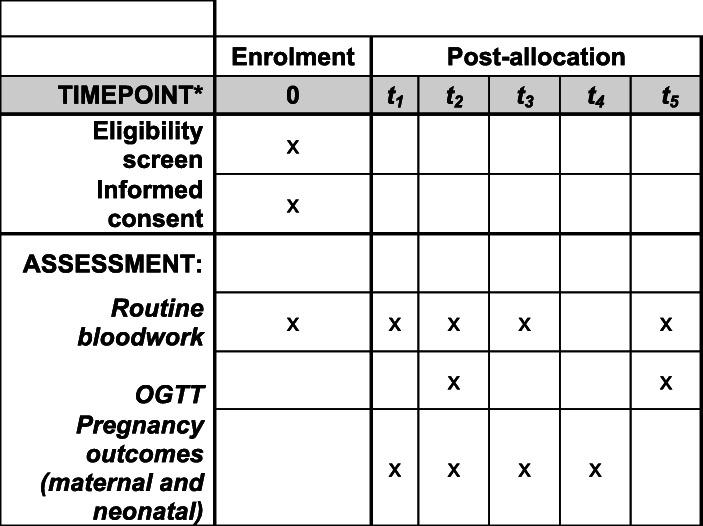
Schedule of enrolment and assessments

Samples for the plasma gCD59 biomarker (one additional EDTA bottle) will be taken together with routine bloods at the first antenatal visit, at weeks 24–28 at the time of routine 75 g OGTT, in trimester 3- and 12-weeks post-partum for women with GDM while having their routine post-partum 75 g OGTT. This means we will have a sample from each trimester of pregnancy and, for women diagnosed with GDM, a sample 3 months post-partum. This will give us the possibility of looking at the predictability of gCD59 in determining GDM in early pregnancy (the trimester 1 sample), mid-pregnancy (the trimester 2 sample, same time as the OGTT), determining pregnancy associated complications ( trimester 1, 2 or 3 samples) or determining the development of glucose intolerance post-partum (the 12 weeks post-partum sample). We will also be able to establish gestational reference intervals for gCD59 in women who do not develop GDM. A schematic schedule of enrolment and assessments can be found in Fig 1.

Each gCD59 plasma sample taken will be aliquoted into 2 × 500 µl aliquots and stored at -80^0^ C. The samples will be barcoded. One aliquot will be analysed for gCD59 levels while the other aliquot will form our biobank and be stored for future analysis. A clinical database linked to the barcoded samples will be developed by the applicant and pseudo-anonymised. This data will be encrypted, password protected and kept on a secure server. DB, POS, and FD will have the key to de-anonymise the data. All laboratory specimens will be identified by a coded ID number to maintain participant confidentiality. To ensure confidentiality, data accessible to team members will be blinded of all identifying participant information.

The constructed database will contain baseline clinical information and laboratory data on each patient, follow-up laboratory data and pregnancy (maternal and neonatal) related outcomes. We will collect neonatal outcomes ordinarily associated with excess glucose (macrosomia, large for gestational age (LGA), hypoglycaemia, shoulder dystocia and birth injuries, respiratory distress, prematurity, death, NICU admission) maternal outcomes associated with excess glucose (polyhydramnios and post-partum haemorrhage (PPH)), maternal outcomes indicative of the greater vascular risk associated with GDM (pregnancy induced hypertension (PIH), pre-eclampsia (PET)), neonatal outcome often associated with disordered placental vascular architecture e.g. small for gestational age (SGA).

LGA is defined as an infant birth weight greater than or equal to the 90th percentile on a standard growth chart and macrosomia as an infant birth weight greater than or equal to 4000 g. SGA is defined as an infant birth weight less than or equal to the 10th percentile for gestational age on a standard growth chart. Neonatal hypoglycaemia is defined as a plasma glucose level of less than 1.65 mmol/L (30 mg/dL) in the first 24 h of life and less than 2.5 mmol/L (45 mg/dL) thereafter.

Prematurity and severe prematurity are defined as a baby born alive before 37 or before 28 completed weeks of pregnancy respectively. Mortality includes stillbirth and neonatal death. Pre-eclampsia is defined as new onset systolic blood pressure (SBP) of at least 140 mmHg and/or diastolic blood pressure (DBP) of at least 90 mmHg at more than 20 weeks’ gestation with proteinuria of greater than 300 mg/day. PIH is defined as new-onset BP at least 140/90 mmHg after 20 weeks gestation with no proteinuria. The decision to proceed with a caesarean delivery is made by the woman’s obstetrician. Polyhydramnios is diagnosed when the amniotic fluid index measured is greater than 24 cm on foetal ultrasound on one or more occasion. Shoulder dystocia is described as foetal shoulders not delivering after the head has emerged from the mother’s introitus due to either one or both shoulders becoming impacted against the bones of the maternal pelvis.

This data will be encrypted, and password protected.

### Ethics

Ethical approval has been obtained from the Galway Clinical Research Ethics Committee. This study is conducted in accordance with the guidelines of the Declaration of Helsinki and Good Clinical Practice.

### Power calculation and sample size

The Obstetrics Division of GUH delivers approximately 3,000 infants each year, of which we conservatively expect to recruit 1,000 per year for a total of 2,000 over the project period. With a GDM prevalence of 12–13% [24], we will have 240–260 subjects who meet OGTT criteria for GDM in the standard of care testing at week 24–28 and who will have had a plasma gCD59 measurement in the first trimester. If we conservatively assume a dropout rate of 5–10%, we will have ≈ 1,800-1,900 subjects who will undergo measurement of plasma gCD59 in the first trimester including 216–230 potentially diagnosed with GDM in the second trimester and having a further plasma gCD59 measurement.

At GUH, approximately 3% of women with GDM develop T2DM in the early postpartum period, an additional 8% develop IFG and 4.7% develop IGT [25]. Based on reported plasma gCD59 values and standard deviation in pregnant women, we estimate that including in the final analysis ≈ 216–230 (potentially > 240) first trimester samples from women later diagnosed with GDM at weeks 24–28 (as per standard of care practices) will provide > 80% power to accurately assess the performance of the plasma gCD59 test in early pregnancy to identify women at risk of GDM, with a precision fixed at 0.05%. We will also have > 80% power to assess the accuracy of plasma gCD59 measured in the second trimester (T2) concomitantly with standard of care OGTT to predict the diagnosis of GDM and to develop an analysis of repeated measurements to assess preliminarily the effectiveness of treatment in women with GDM. Also, in a cohort of 215–230 women with a diagnosis of GDM, at a postpartum conversion rate to type 2 diabetes of 3% with additional ≈ 13% converting to glucose intolerance (Impaired Fasting Glucose (IFG) and Impaired Glucose Tolerance (IGT)[25], we expect to have 34–37 women who will develop either diabetes or pre-diabetes in the early post-partum period. If postpartum (6–12 weeks) levels of plasma gCD59 are comparable to those reported in a non-pregnant population, we expect to have 80% power to identify women with GDM who convert to glucose intolerance after delivery.

### Statistical Analysis

Patient characteristics will be described using mean and standard deviations/ medians and interquartile ranges for continuous variables (depending on data distribution) and count proportions for categorical variables. The bivariate baseline characteristics of GDM cases and controls will be compared using binomial/multinomial logistic regression with adjustment for covariates. The sensitivity and specificity of plasma gCD59 to predict the results of the OGTT will be assessed using nonparametric estimates of the receiver operating characteristic (ROC) curves and respective area under the receiver operating characteristic (ROC) curve (AUROC) [26]. The level for significance for all tests conducted will be set at α = 0.05, with all reported *P* values as two-tailed. Multiple imputation will be used for missing data.

### Study Status

Recruitment has started February 2019 and it is estimated it will take 18 months for full recruitment.

## Discussion

A body of clinical and experimental evidence supports a link between the complement system, complement regulatory proteins, and the pathogenesis of diabetes complications [27–31].

CD59 is a cell membrane-bound protein. However, a soluble form of CD59 that is shed from cell membranes by phospholipases is present in human blood, urine, saliva, and other body fluids [32–34]. In diabetes, non-enzymatic glycation inactivates the complement inhibitor CD59, forming glycated CD59. By using a highly sensitive and specific ELISA assay, levels of gCD59 were found to be 3- to 4-fold higher in individuals with type 2 diabetes, higher gCD59 concentrations were strongly associated with higher glucose levels after 2-hour oral glucose tolerance tests and the gCD59 level has also been shown to acutely parallel changes in glycaemic control during therapeutic intervention with insulin [35, 36]. Furthermore, in a population screened using a GCT median gCD59 levels were 8.5-fold higher in the 500 case patients that failed the GCT compared to the control subjects and 10-fold higher in the 127 case patients in whom GDM was diagnosed by the subsequent 3-h OGTT. In a recent retrospective study [37], gCD59 was found to be an accurate biomarker for the early prediction of GDM (AUROC = 0.90) and also plasma levels of gGD59 were positively associated with the risk of infant malformations, neonatal hypoglycaemia or delivering an LGA baby (Ref). However, one of the main limitations of this study was that the population recruited was very selective with a BMI > 29 kg/m^2^.

Building on this research, our study plans to look at the gCD59 capacity to classify pregnant women with normal or abnormal glucose tolerance as defined by the 2-hour, 75-g OGTT recommended by the IADPSG criteria in an Irish cohort but also assess if gCD59 can be used as an early predictor for GDM, a predictor for adverse pregnancy outcomes and/or post-partum glucose intolerance.

The results will be accessible to physicians and patients and will be published in peer reviewed international literature journals.

## Data Availability

The anonymised datasets used and/or analysed during the current study will be available from the corresponding author on reasonable request.

## Abbreviations

BMI: Body Mass Index
DAF: Decay Accelerator Factor
DBP: Diastolic Blood Pressure
GDM: Gestational Diabetes
GCT: Glucose Challenge Test
HSE: Health Service Executive
IADPSG: International Association of the Diabetes and Pregnancy Study Groups
IFG: Impaired Fasting Glucose
IGT: Impaired Glucose Tolerance
LGA: Large for Gestational Age
MAC: Membrane Attack Complex
MCP: Membrane Cofactor Protein
NICU: Neonatal Intensive Care Unit
OGTT: Oral Glucose Tolerance Test
PET: Pre-eclampsia
PIH: Pregnancy Induced Hypertension
PPH: Post-partum haemorrhage
SGA: Small for Gestational Age
SBP: Systolic Blood Pressure
